# Analyzing captive breeding outcomes to inform reintroduction practice: lessons from the pookila (*Pseudomys novaehollandiae*)

**DOI:** 10.1093/jmammal/gyad056

**Published:** 2023-06-21

**Authors:** Kiarrah J Smith, Maldwyn J Evans, Iain J Gordon, Jennifer C Pierson, Jenny Newport, Adrian D Manning

**Affiliations:** Fenner School of Environment and Society, The Australian National University, Acton, Australian Capital Territory 2601, Australia; Fenner School of Environment and Society, The Australian National University, Acton, Australian Capital Territory 2601, Australia; Department of Ecosystem Studies, Graduate School of Agricultural and Life Sciences, The University of Tokyo, Tokyo 113-0032, Japan; Fenner School of Environment and Society, The Australian National University, Acton, Australian Capital Territory 2601, Australia; The James Hutton Institute, Dundee DD2 5DA, United Kingdom; Central Queensland University, Townsville, Queensland 4810, Australia; Land and Water, CSIRO, Townsville, Queensland 4810, Australia; Lead, Protected Places Mission, National Environmental Science Program, Reef and Rainforest Research Centre, Cairns, Queensland 4870, Australia; Fenner School of Environment and Society, The Australian National University, Acton, Australian Capital Territory 2601, Australia; Australian Wildlife Conservancy, Subiaco East, Western Australia 6008, Australia; Centre for Conservation Ecology and Genomics, Institute for Applied Ecology, University of Canberra, Canberra, Australian Capital Territory 2617, Australia; Fenner School of Environment and Society, The Australian National University, Acton, Australian Capital Territory 2601, Australia; Fenner School of Environment and Society, The Australian National University, Acton, Australian Capital Territory 2601, Australia

**Keywords:** captive breeding, conservation, reintroduction, reproduction, rodent, translocation tactics

## Abstract

Captive breeding is often used to produce individuals for reintroduction programs in order to reestablish a species in an area where it has become locally extinct. To maximize the likelihood of establishing a self-sustaining population in the wild, an analysis of data from captive breeding programs is commonly undertaken to (1) increase the *quantity* of individuals and rate at which they can be released, and (2) maintain or improve the genetic and phenotypic *quality* of individuals. Here we demonstrate how the knowledge gained from these analyses can also be applied to decision-making during the design of subsequent reintroductions to further advance a reintroduction program toward success. We conducted an analysis of data from a captive breeding program for the threatened pookila (*Pseudomys novaehollandiae*, New Holland mouse) spanning 6 years. We found evidence for relationships between the reproductive output of pookila and behavioral, demographic, experiential, health, and physiological predictors. Based on a biological interpretation of these results, and with reference to a checklist of all known translocation tactics, we recommend 11 specific design elements to maximize the probability of pookila reproduction postrelease (thereby improving the likelihood of reintroduction success). These recommendations should be interpreted as hypotheses to be evaluated and refined in future reintroduction trials for the pookila. The uncertainty around the postrelease survival and reproduction of a species that is common in reintroduction practice warrants the creative use of existing data to inform adaptive management. Indeed, there is a wealth information in well-kept captive breeding records that is currently underused by reintroduction practitioners. The direct integration of knowledge derived from captive breeding (where available) with decision-making for reintroductions, as described here, will help navigate these uncertainties, which would benefit the conservation of both understudied and well-known species around the world.

## Teaser

The direct integration of knowledge derived from captive breeding with decision-making for reintroductions can help navigate uncertainties around postrelease survival and reproduction, offering benefits for the conservation of both understudied and well-known species.

A species reintroduction program is a long-term endeavor that begins with the collection of animals from a wild source population and ends with the establishment of a self-sustaining population in an area where the species went locally extinct (IUCN/SSC 2013; Evans et al. 2022). Intermediary steps in this ‘pathway to the wild’ (sensu Evans et al. 2022) take the form of trials and experiments undertaken within an adaptive management framework across a range of settings, including in captivity and inside predator-proof safe havens (Batson et al. 2015; Evans et al. 2022). Evidence from a range of trials has shown that a reintroduction is more likely to succeed if there is a large number of founders released or available for regular reinforcements (Hardy et al. 2018; Morris et al. 2021). However, if the number of founders is insufficient, and/or the risk of negative impacts on remnant populations is too high to collect more from the wild, captive breeding is the necessary alternative (Hogg 2013).

Knowledge gained through the analysis of data from captive breeding programs is commonly applied to (1) increase the *quantity* of individuals and rate at which they can be released, and (2) maintain or improve the genetic and phenotypic *quality* of individuals (i.e., suitability for release to the wild). An evidence-based adaptive approach to husbandry modifications for the purpose of increasing breeding efficiency (e.g., likelihood of pairing success, litter size, or juvenile survival) is already well-established among zoos and other captive breeding facilities (Smith et al. 2011; Saunders et al. 2014; Little et al. 2016; Bauman et al. 2019; Lemasson et al. 2020). The main concern regarding the quality of individuals for reintroductions is the potential for negative genetic and phenotypic changes from the ‘wild type,’ such as inbreeding depression and adaptation or habituation to captivity, which can have impacts on fitness and cause maladaptation to the wild (Schwartz and Mills 2005; Frankham 2008; Slade et al. 2014). For example, the population-level variability in predator-response behavior of oldfield mice (*Peromyscus polionotus subgriseus*) has been observed to increase with generations in captivity (McPhee 2004). Consequently, oldfield mice from later generations were less likely to remain under cover in the minute immediately following exposure to an artificial predator stimulus (McPhee 2004). Methods used to mitigate potential impacts on the quality of individuals for reintroduction include minimizing the number of generations in captivity, supplementing genetic diversity with additional wild founders, and providing conditions and enrichment similar to the wild environment (Williams and Hoffman 2009). There is also increasing interest in *improving* the quality of individuals by enhancing their capacity to coexist with evolutionarily novel threats in the wild through prerelease training (e.g., predator aversion training and controlled predator exposure; (Evans et al. 2022; Taylor et al. 2022) or breeding individuals with advantageous traits (e.g., targeted gene flow; Kelly and Phillips 2019).

**Fig. 1.**
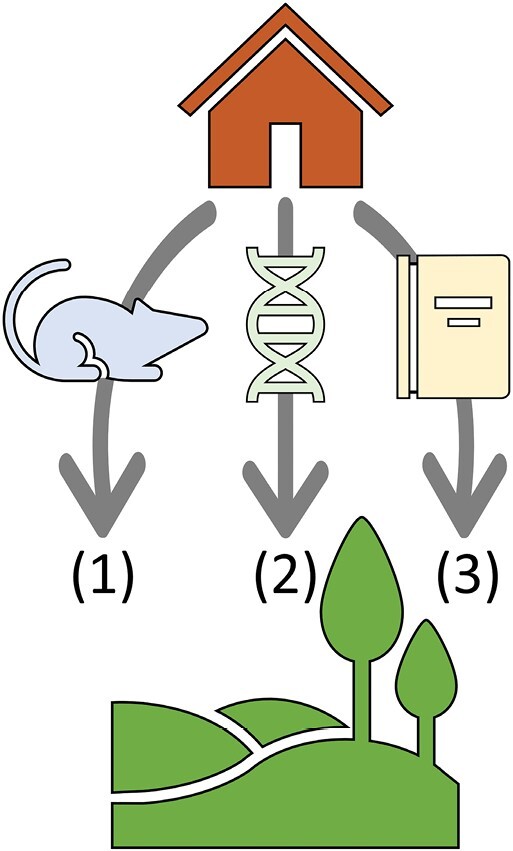
There are three ways the analysis of captive breeding outcomes (house) may advance a reintroduction program toward success (landscape): (1) improving breeding efficiency to increase the *quantity* of individuals and rate at which they can be released; (2) maintaining or improving the *quality* of individuals by minimizing genetic and phenotypic change from the wild type, training individuals, or breeding individuals with advantageous traits; and (3) the direct application of the knowledge gained from those analyses to decision-making during the design of a reintroduction.

The direct application of knowledge gained from the analysis of captive breeding outcomes to decision-making during the design of subsequent reintroductions (Fig. 1) has received relatively little attention. Here, we demonstrate the value of this application by conducting an analysis of data from a captive breeding program for the Australian native pookila (*Pseudomys novaehollandiae*, New Holland mouse). Reintroductions are a priority for the conservation of the pookila because it is currently vulnerable and experiencing a fragmented and decreasing population (Woinarski and Burbidge 2016; Wilson et al. 2018; Lazenby et al. 2019; Burns 2020). Research surrounding previous reintroductions of the pookila has included: trials of soft-release enclosures and monitoring techniques (Wilson et al. 2003, 2018); an examination of habitat selection and changes in the genetic diversity of pookila through the reintroduction process (Abicair et al. 2020); and the development of “Mini Safe Havens” (i.e., permanent semipermeable refuge areas) to facilitate the in situ learning and adaptation of pookila to coexist with key threats (Smith et al. 2022). Experimental translocations have also been undertaken to examine the recolonization of regenerating habitat by pookila, and the potential for competition with the introduced house mouse (i.e., laboratory mouse, *Mus musculus*; Fox and Pople 1984; Fox and Twigg 1991). However, there remains a high level of uncertainty around the postrelease reproductive output of the species. Such uncertainty warrants the creative use of existing data to inform adaptive management (McCarthy et al. 2012). Our primary goal was to demonstrate how an analysis of captive breeding data can be used to make recommendations for the design of a successful reintroduction. The intent is that these recommendations should be interpreted as hypotheses to be tested in future reintroduction trials within an adaptive management framework (Batson et al. 2015). In this case, the aim was to maximize the probability of pookila successfully reproducing postrelease (thereby improving the likelihood of reintroduction success).

The pookila belongs to an evolutionary distinct group of endemic Australian rodents (Rowe et al. 2008) and has a relatively slow reproductive rate compared to other rodent groups (Geffen et al. 2011). Although the myriad of studies for the house mouse can aid the biological interpretation of our analysis, it is possible that the factors affecting the reproductive output of pookila differ from what is known for house mice and other rodent species.

## Materials and Methods

### Captive colony.

We started the pookila captive breeding colony at the Australian National University (ANU) in 2016. A total of 21 wild founders were sourced from Nabiac Water Treatment Plant and the nearby Booti Booti National Park within the traditional lands of the indigenous Worimi and Biripi peoples in the state of New South Wales (Table 1). At a regional scale, these two localities are considered to form part of the same population of pookila. In 2018, we added two females to the colony, also sourced from land at Nabiac Water Treatment Plant. Based on the absence of active or regressed teats, all females collected from the wild were believed to be in their first year. Our husbandry protocol was developed over time, drawing from previous pookila colonies (Kemper 1976a, 1976b; Prosser et al. 2007), studies of wild populations (Kemper 1980; Wilson and Bradtke 1999), expert advice, and our own observations. We made several protocol modifications to accommodate efficient husbandry with the ultimate goal of supporting successful reproduction. The main challenges we needed to overcome were excessive weight gains, high daytime activity levels (pookila are a nocturnal species), and aggression during pairing. All procedures were approved by the ANU Animal Experimentation Ethics Committee (protocols A2014/42, A2017/31, and A2020/30) and followed the American Society of Mammalogists guidelines for research on live animals (Sikes et al. 2016).

**Table 1.** Spatial and temporal collection details for the 23 pookila (*Pseudomys novaehollandiae*) founders sourced across Nabiac Water Treatment Plant (NW) and Booti Booti National Park (BB) for the captive breeding colony at the Australian National University. The “Progeny?” column indicates whether the individual was a true founder (“True”) or never successfully contributed progeny to the colony (“False”). A dash (–) indicates missing data.

Locality	Session	Point	Sex	Progeny?
BB	February 2016	S1	M	True
BB	February 2016	S5	F	False
BB	February 2016	S7	M	False
BB	February 2016	S7	F	True
BB	February 2016	S8	M	False
BB	February 2016	S8	F	True
BB	February 2016	S10	M	False
BB	July 2016	H20	F	False
BB	July 2016	N13	M	True
BB	July 2016	N13	F	True
BB	July 2016	S1	F	True
BB	July 2016	S5	F	False
BB	July 2016	S6	F	False
BB	July 2016	S7	F	True
BB	July 2016	S10	M	True
BB	July 2016	S14	M	True
BB	July 2016	S14	F	True
BB	July 2016	S15	M	True
NW	June 2016	C2	F	True
NW	June 2016	C5	M	True
NW	June 2016	C7	F	True
NW	March 2018	C2	F	True
NW	March 2018	–	F	True

The distance between the collection points of male and female founders (Table 1) was considered in our initial pairing protocol in order to decrease the likelihood that pairs were close relatives (Gauffre et al. 2008). However, over time, pairing decisions became more random as we sought to create compatible pairs. The genetic relationships of the founders were unknown and we assumed that they were unrelated and not inbred, as is conventional for pedigree-based management of captive breeding programs (Rudnick and Lacy 2008). Subsequently, we used a handwritten pedigree to avoid pairing known relatives. This method became unsustainable as the population grew (and inadvertently resulted in nine pairings of relatives equal to half-siblings or greater), so in late 2019, we digitized the pedigree to a Poplink studbook (Faust et al. 2019). Thereafter, our pairing protocol was based on a minimum Mate Suitability Index (MSI), which is a composite score for the value of a pairing calculated on the complete pedigree by the population management software PMx (Traylor-Holzer 2011; Lacy et al. 2012). We made some “slightly detrimental” (MSI = 4) and “detrimental” (MSI = 5) pairings because our priority was to produce a large number of animals for reintroductions, and a supplementation of additional wild founders was not possible at the time. Other pairing parameters variously incorporated, adjusted, or removed from the protocol included: selecting for minimum weight difference between the male and female; a minimum and maximum age and weight for inclusion in breeding rotation; a maximum number of litters before retirement from breeding; the exclusion of individuals with health conditions; and avoiding multiple pairings of the same male and female. Although pookila have a postpartum estrus and have been successfully housed continuously with a breeding partner (Kemper 1976b), we did not pursue this practice beyond the first few months in the ANU colony because there were frequent aggressive interactions and we wanted the females to have a respite between litters. Instead, we introduced a male to a female enclosure for a number of days, and then separated them before the earliest possible parturition date. The first litter of pups was born 6 months after the colony commenced. Ultimately, 16 of the founders (10 female, 6 male) bred successfully (Table 1). This outcome reflects the challenges associated with establishing ideal environmental conditions for pookila to breed in captivity, which may have exerted inadvertent selection against individuals that required more natural conditions to reproduce.

### Data.

We used data spanning seven generations from 14 February 2016 to 4 April 2022 for our analysis (371 pairings with complete data, of which 182 included at least one wild-born parent). Our hypothesis was that the reproductive output of pookila (Table 2) was influenced by behavioral, demographic, experiential, pedigree, health, and physiological factors (Table 3). These categories reflected relevant groupings for animal-focused tactics in the Translocation Tactics Classification System (Batson et al. 2015). This framework is a comprehensive checklist of tactics that can be applied to reintroductions to aid decision-making, which has been proven to be highly effective in improving reintroduction success when implemented with an adaptive management approach over successive trials (Wilson et al. 2020).

**Table 2.** Five response variables related to reproductive success that we chose as metrics of reproductive output for the pookila (*Pseudomys novaehollandiae*) breeding colony at the Australian National University.

Response	Description with summary statistics
Pairing success (PS)	Presence (22%) or absence of pups, including pregnancies ending in stillbirth or other complications, but none where embryos might have been resorbed (Chung et al. 2018).
Aggression (AG)	Observed presence (18%) or absence of injury to one or both mice during pairing. Aggression was also included as a predictor (Table 3).
Litter size (LS)	Number of pups in a litter ($\bar{x}$ = 3, range = 1–6, *n* = 81). Excludes pups that may have been consumed by the mother in the first few days after parturition (Gustafsson et al. 1980).
Sex ratio (SR)	Proportion of male pups in a litter ($\bar{x}$ = 0.5 ± 0.3 *SD*, *n* = 81), weighted by litter size.
Breeding complications (BC)	Of pairings where pups were present (*n* = 81), presence (10%) or absence of death during pregnancy (confirmed by postmortem) or parturition, or death of pups prior to weaning (at 28 days old).

**Table 3.** Predictor variables considered for the analysis of five response variables related to the reproductive output of the pookila (*Pseudomys novaehollandiae*) in the breeding colony at the Australian National University: pairing success (PS), aggression (AG), litter size (LS), litter sex ratio (SR), and breeding complications (BC) (see Table 2). A tick (✓) indicates a variable that was included in the final model for the relevant response variable. A cross (✘) indicates a variable included in the initial analysis, but removed from the final model due to correlation detected after scaling the input variables ^‘C’^, or multicollinearity observed after initial modeling (high ^‘H’^, or moderate ^‘M’^). A dash (–) indicates variables that were not considered applicable to the relevant response variable and therefore not included in analysis. *F. health was considered important for BC but not included since no breeding complications occurred for females that had health conditions. Where reported, summary statistics are means ± *SD*.

Predictor	Description	PS	AG	LS	SR	BC
Behavioral						
Aggression	Observed presence or absence (base level) of injury to one or both mice during pairing. Aggression was also included as a response (Table 2).	✓	–	✓	✓	✓
Demographic						
F. age	Female age in days when the pair was made. The birthday of wild-caught founders was estimated to be the middle of the breeding season prior to capture (15th December).	✓	✓	✓	✓	✓
M. age	Same as “F. age,” but for males.	✓	✓	–	✓	–
Abs. age dif.	Absolute difference between male and female age at pairing.	✓	✓	–	–	–
F. gen.	Female generation number (Pollak 2008) where the wild-caught founders were generation 1. Offspring generation was assigned as the generation of the most recent parent plus 1 (Farquharson et al. 2017).	✓	✓	✓	✓	✘^M^
M. gen.	Same as “F. gen.,” but for males.	✘^C^	✘^C^	–	✘^M^	–
Experiential						
F. origin	Wild (base level) or captive-bred origin for the female (Farquharson et al. 2018).	✓	✓	✓	✓	✓
M. origin	Same as “F. origin,” but for males.	✓	✓	–	✓	–
F. exp.	Count of times a female was paired, including the pairing in question.	✘^C^	✘^C^	✘^C^	✘^C^	✘^C^
M. exp.	Same as “F. exp.,” but for males.	✘^C^	✘^C^	–	✘^C^	–
F. first pair	Age at which the female was first paired (Rosenfeld et al. 2003; Farquharson et al. 2017).	✓	✓	✓	✓	✓
Pair type	The pairing of a particular male and female has never been made before (“new,” base level); has been made previously and was successful (“effective”); or has been made previously but with no success (“failed”; Bauman et al. 2019).	✓	✓	✓	✓	–
Days paired	Number of days the pair were housed together.	✓	✓	✓	✓	–
Sim. pairs	Number of pairings in the same room concurrent for any number of days, including the pairing in question (Asaba et al. 2014).	✓	✓	✓	✓	–
Pups pres.	Presence or absence (base level) of pups (<28 days old) for any period during pairing.	✓	✓	✓	✓	–
Pedigree						
F. inb.	Female’s inbreeding coefficient (Fredrickson et al. 2007) estimated from the pedigree using PMx (Lacy et al. 2012) with the assumption that founders were unrelated and not inbred (*n* = 371, $\bar{x}$ = 0.003 ± 0.017; *n* = 81, $\bar{x}$ = 0.007 ± 0.026). Equivalent to the kinship coefficient of the individual’s parents (Traylor-Holzer 2011).	✓	✓	✓	✓	✓
M. inb.	Same as “F. inb.,” but for males (*n* = 371, $\bar{x}$ = 0.003 ± 0.016; *n* = 81, $\bar{x}$ = 0.006 ± 0.025).	✓	✓	✓	✓	✓
Kinship	Kinship coefficient of the pair calculated in PMx based on the pedigree with the assumption that founders were unrelated and not inbred (*n* = 371, $\bar{x}$ = 0.011 ± 0.030; *n* = 81, $\bar{x}$ = 0.015 ± 0.027). Equivalent to the inbreeding coefficient of offspring that could be produced (Traylor-Holzer 2011).	✓	✓	✓	✓	✓
Health						
F. health	Presence or absence (base level) of seizures or a metabolic condition in the female. The metabolic condition was present in one female and was characterized by obesity, an inability to lose weight despite diet restrictions, and frequent urination. Seizures were recorded when an individual displayed involuntary shaking after handling (Pesapane and Good 2009). However, we note there was inconsistency in our early definition, which means that some potentially natural “freeze” behaviors (where the animal stays still in response to a threat or stress; Rabi et al. 2017) were recorded as seizures.	✓	✓	–	–	–*
M. health	Presence or absence (base level) of health conditions (seizures, freezing episodes—see “F. health”) in the male.	✓	✓	–	–	✓
Physiological						
F. parity	Number of times the female had previously given birth: nulliparous (base level), primiparous, or multiparous (Bauman et al. 2019).	✓	✓	✓	✓	✓
M. pat.	Number of times the male had previously fathered pups: nulliparous (base level), primiparous, or multiparous (Edwards and Cameron 2014).	✓	✓	–	✓	✓
F. weight	Most recently recorded weight for the female when the pair was made (generally recorded within 2 weeks of pairing).	✓	✓	✓	✓	✓
M. weight	Same as “F. weight,” but for males.	✓	✓	–	✓	–
Abs. wt. dif.	Absolute percent difference between male and female weight.	✓	✓	–	–	✓
Diet	High-fat (base level): variously including pellets ad libitum, sunflower seeds, fruit, meal worms, and raw nuts. Low-fat: measured amounts of seed, pellets, vegetables, and legumes, no high-fat or high-sugar inclusions except a peanut given under certain conditions; implemented from 30th August 2017. Full: larger portion and variety of the low-fat diet given from the day of pairing to the female’s latest potential parturition date; implemented from 29th June 2021 (Rosenfeld et al. 2003). Note: Any effect of diet may also reflect other changes we made to the husbandry protocol over time (e.g., enhanced lighting, provision of grass), for which we have less certainty about which pairings they were applied to.	✘^M^	✘^M^	✘^M^	✘^H^	✓

We chose the input variables based on studies of other species, as well as hypotheses arising from our observations of the colony (Table 3). We did not make assumptions about what might reasonably influence the reproductive output of pookila. However, our scope was constrained by the data that had been recorded for the colony (e.g., no genetic data were available for the colony at the time of writing).

### Analysis.

**Fig. 2.**
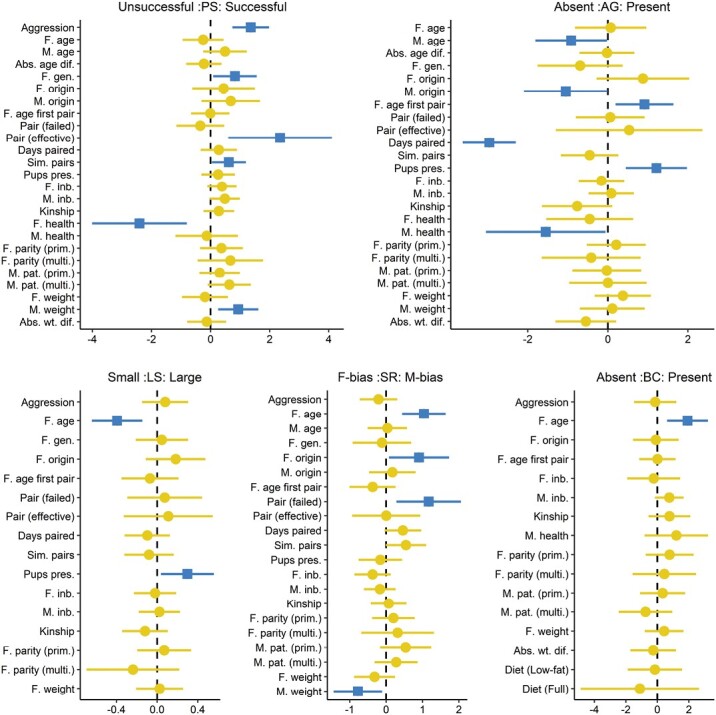
Estimate and 90% confidence interval from the conditional model-averaged coefficients for all variables included in the final models of pairing success (PS), aggression (AG), litter size (LS), sex ratio (SR), and breeding complications (BC) for the pookila (*Pseudomys novaehollandiae*). Input variables were standardized following Gelman (2008), so the effect sizes for continuous and binary variables are on a comparable scale. Effects with a square point had at least weak evidence (i.e., a *P*-value of ≤0.1) of a relationship between the predictor and response variable (Muff et al. 2022). Strength of evidence increases with the distance of the confidence interval from the midline. There was little to no evidence for a relationship with the response variable for the predictors that have a circular point. Base levels for factor variables are listed in Table 3.

To determine which of the variables influenced reproductive output, we used a model averaging procedure on a fitted series of generalized linear (GLM) and generalized linear mixed models (GLMM) for each response variable using the ‘lme4’ (Bates et al. 2015) and ‘brglm2’ (Kosmidis 2021) packages in R (R Core Team 2022). We assumed a Poisson error distribution with a log-link for the litter size (LS) response variable (count) and binomial error distribution with a logit-link for the other (binomial) response variables. For each response variable, we created a subset of the data that included only the input variables and pairings we considered applicable to the response variable in question (Table 3). Pairing success (PS) and aggression (AG) analyses used all pairings with complete data (*n* = 371), which excluded four accidental sibling matings and seven pairings where the outcome could not be certain (e.g., death of a female where autopsy found no evidence of pregnancy). Analysis of the other three response variables used successful pairings only (*n* = 81, accidental sibling matings excluded), with the sex ratio (SR) data set further subsetted to remove eight cases where pup sexes were unknown. Then, to enable the direct comparison of coefficients resulting from continuous and binary predictors, we rescaled all continuous input variables to a mean of 0 and standard deviation of 0.5, centered binary factors to a mean of 0, and left all factors with more than two levels unchanged (Gelman 2008; Gelman and Su 2021). Any correlations between continuous variables greater than 0.6 or less than −0.6 were remedied by removal of the least biologically relevant variable of the pair (Grueber et al. 2011). To account for repeated measures of the same pookila, we included individual male and individual female identification codes as random factors. In the case of the breeding complications (BC) response variable, which demonstrated complete separation (i.e., where maximum likelihood estimates tend to infinity; Firth 1993; Heinze and Schemper 2002), we used bias reduction (Kosmidis 2021), which is not yet implemented for mixed models (i.e., requiring GLM rather than GLMM). In all fitted models, we checked for multicollinearity using the ‘performance’ package (Lüdecke et al. 2021) in R and removed one variable at a time (starting with the least interesting or biologically relevant variable with high correlation) and reran the model until only variables with low correlation remained (Table 3). We then fitted all subsets of this final full model (Supplementary Data SD1) and performed model averaging on all models below ∆AICc = 6 (Burnham and Anderson 2002; Anderson 2007) using the ‘MuMIn’ package (Bartoń 2022). We plotted the conditional model-averaged coefficients (Supplementary Data SD2) as effect size plots (Wickham 2016). For each of the predictors that showed at least weak evidence of a relationship with reproductive output (Fig. 2; Supplementary Data SD2), we plotted predictions and unconditional standard error computed from the model set below ∆AICc = 6 (function: ‘modavgPred’; Mazerolle 2020) or in the case of breeding complications, the top model (function: ‘predict’; R Core Team 2022). An example R code for each step in our analyses has been made available online (Smith and Evans 2022). Results are interpreted in terms of a continuous spectrum from little (or none) to very strong evidence, which reflects the gradual process of knowledge gathering and adaptive management better than the traditional ‘black-and-white’ null-hypothesis significance testing (Muff et al. 2022).

## Results

All of the variables included in the final models were retained through model averaging (Fig. 2). Variables that are missing (e.g., diet) are those removed due to correlation or multicollinearity before modeling (Table 3).

### Behavioral predictors.

**Fig. 3.**
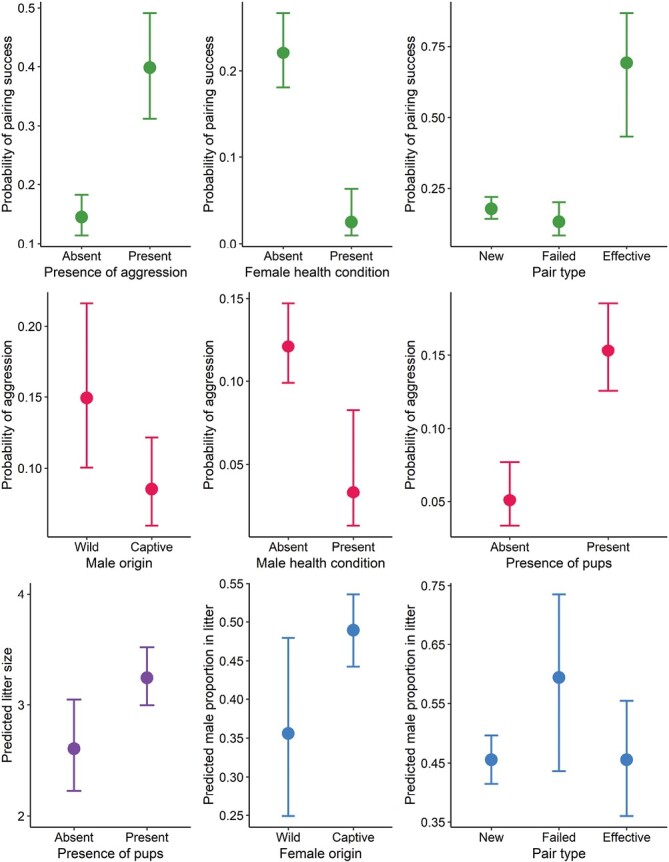
Predicted values (points) with standard error (bars) for each factor variable where there was at least weak evidence of a relationship with pairing success, aggression, litter size, or sex ratio (Fig. 2) for the pookila (*Pseudomys novaehollandiae*). In all cases, the category on the left is the base level. The degree of difference between the categories increases with decreasing overlap of the standard errors.

The observation of signs of aggression during pairing corresponded to a ~40% chance that pups would be produced, which was much greater than the ~14% chance of success when no aggression was recorded (Fig. 3). There was no evidence that aggression influenced any of the other metrics of reproductive output (Fig. 2, Supplementary Data SD2).

### Demographic predictors.

**Fig. 4.**
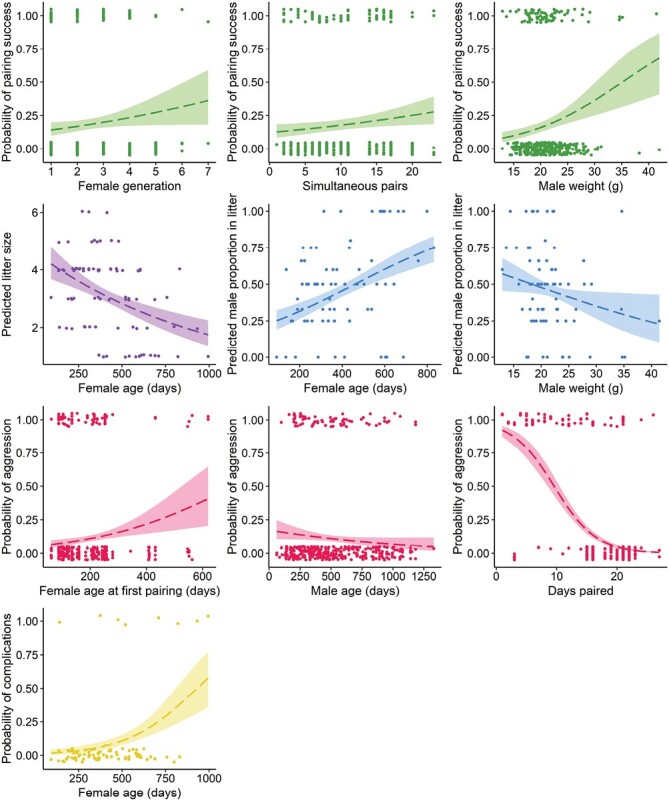
Predicted values with standard error bands for each continuous variable where there was at least weak evidence of a relationship with pairing success, litter size, sex ratio, aggression, or breeding complications (Fig. 2) for the pookila (*Pseudomys novaehollandiae*). Strength of the relationship is highest at the narrow parts of the error bands. We plotted raw data points with a “jitter” (width = 0, height = 0.05), except in the sex ratio plots, where no jitter was applied.

The likelihood of successful pairing increased with female generation number, but became more uncertain after generation 4 (Fig. 4). Among successful females, those that were older were more likely to die during pregnancy or parturition, or have their pups die prior to weaning (i.e., ~27% risk of breeding complications at 2 years old; Fig. 4). Older females also produced smaller litters that tended to be male-biased (Fig. 2). The probability that signs of aggression would be observed during pairing was typically below 10% when the male was older than ~600 days (Fig. 4).

### Experiential predictors.

There was a positive relationship between the probability of pairing success and the number of simultaneous pairings undertaken in the same room (Fig. 4). Additionally, a male/female pair that had previously produced pups together was much more likely to be successful again than a new pairing, or a previously unsuccessful pairing (Fig. 3). A pairing that was previously unsuccessful was more likely to produce a male-biased litter than was a new pairing (Fig. 3). Compared to the female-biased litters of wild-born females, captive-born females tended to produce litters closer to a 1:1 sex ratio (Fig. 3).

There was a very strong negative relationship between the probability of aggression and the number of days a male was housed with a female (Fig. 4). Pairs with a captive-born male were less likely to result in signs of aggression than those where the male was wild-born (Fig. 3). Signs of aggression were more likely to be observed as the age at first pairing for the female increased (Fig. 4). When pups from other pairings were present in the room, the probability that aggression signs would be observed for a pairing increased to ~15% (relative to ~5% when pups were absent), and the litter size for a successful pairing was predicted to be larger (Fig. 3).

### Pedigree predictors.

There was little to no evidence for a relationship between our pedigree-derived predictors and any measure of reproductive output (Fig. 2, Supplementary Data SD2).

### Health predictors.

Females known (or later discovered) to be suffering a metabolic condition, seizures, or freezing episodes (see Table 3 for explanation) were much less likely to produce pups than females without a health condition (Fig. 3). There was also some evidence to suggest that signs of aggressive interactions would be absent when the male was suffering a health condition (Fig. 2).

### Physiological predictors.

Heavier males were more likely to sire pups and have female-biased litters than lighter males (Fig. 4). No other effects of physiological predictors were evident (Supplementary Data SD2).

### General observations.

**Fig. 5.**
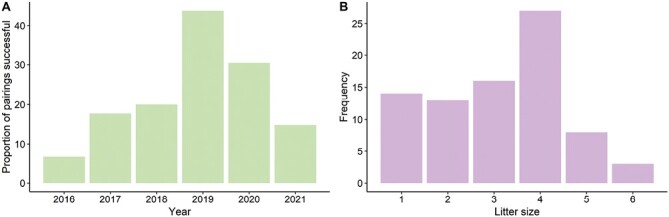
A) The annual pairing success rate for the pookila (*Pseudomys novaehollandiae*) in the Australian National University captive breeding colony increased over time, peaking in 2019. B) Litters of five or six pups were the least common in this population (pookila have only four teats). These litter size frequencies can be incorporated into population viability analyses for the pookila.

Pairing was most successful in 2019 (Fig. 5), where pairings with one wild- and one captive-born parent (*n* = 12) were 42% successful, while pairings where both parents were captive-born (*n* = 36) were 44% successful (no pairings with two wild-born parents were made in 2019). This peak occurred just prior to a bottleneck event caused by the need to drastically reduce colony size from 227 (including 10 wild founders) to 20 individuals (no wild founders were retained), and pause pairing for more than 9 months due to COVID-19 lockdown restrictions in 2020 (i.e., limits imposed on animal technicians flowed on to restrict the capacity of the pookila colony). The youngest sire was an accidental sibling mating that occurred at an estimated age of 108 days, while the youngest female to produce pups was 92 days old at pairing (very few pairings were attempted for younger individuals in the ANU colony). The oldest female to produce pups without breeding complications was 831 days old at pairing, while the oldest successful male born in the colony was 1,153 days old at pairing. A litter size of four was the most common (Fig. 5). Aggressive interactions resulted in injuries to the male more often than to the female (Smith K.J., ANU, Canberra, Australian Capital Territory, personal observation, April 2022).

## Discussion

Our analysis identified a number of factors that did (and did not) influence the reproductive output of pookila in the ANU colony. Each of these results provide crucial information that will aid the conservation of the species by providing a baseline from which practitioners can: (1) improve the efficiency of breeding pookila in captivity; (2) maintain or improve the suitability of captive individuals for release to the wild; and (3) refine release methods to increase the likelihood of reintroduction success. Here we demonstrate the last of these three applications by undertaking a biological interpretation of our results to make tactical recommendations (sensu Batson et al. 2015) for the design of a reintroduction that could maximize the probability of pookila successfully reproducing postrelease (Table 4). We acknowledge that the captive environment may impose different effects on reproductive output than the wild. However, due to the challenges of monitoring ecologically cryptic species with the technology available at the time of writing, we suggest that the use of knowledge gained from the captive environment to guide reintroduction tactics and formulate hypotheses within an adaptive management framework is better than starting from scratch.

**Table 4.** Recommended translocation tactics (sensu Batson et al. 2015) to use (‘Do’), avoid (‘Don’t’), and experiment with (‘Trial’) for the design of a reintroduction that could maximize the probability of pookila (*Pseudomys novaehollandiae*) successfully reproducing postrelease (thereby improving the likelihood of reintroduction success), based on a biological interpretation of results from an analysis of captive breeding data. The hypotheses underpinning these design elements (to be evaluated and refined in future trials) are indicated by superscript letters, defined as follows: A) Minimize the likelihood of aggression in successful pairings. C) Minimize the likelihood of breeding complications. F) Encourage female-biased litters. L) Encourage large litters. N) Unlikely to negatively affect population establishment. P) Promote pairing success.

Tactic	Do	Don’t	Trial
Animal selection			• Release of newly weaned animals^A,F,L^ • Release of individuals that have a health condition^N^
Preconditioning	• Pair individuals from a young age and release after the outcome of at least one pairing is known^A^		• Release of females already mated in an effective pair^P^
Release composition	• For previously successful females: release together with males they are known to be effective with^P^ • For females with no previous pairing success: provide free mate choice^P^	• Limit release to entirely old individuals^C,F,L^	
Resource augmentation	• Provide space and structural complexity at the release site^A^		• Provision of bedding from boxes containing breeding pairs, pregnant females or pups^L,P^
Release configuration			• Various holding periods to maximize site fidelity while minimizing risk of injuries, which may include hard release^A,P^
Postrelease management		• Feed high-fat supplementary foods for long periods^P^	

### Provide space and structural complexity.

The strong correlation between a relatively long pairing duration and the absence of injuries caused by aggression reinforces the importance of following our current protocol to separate a pair early if more than three bites on the tail or one wound on any other body part is detected. Indeed, the occurrence of aggression-related injuries can be taken as an indicator that a pairing will be successful, rather than a sign of incompatibility. However, the presence of aggression does not guarantee pairing success (i.e., one should not aim to cause aggression). Since pookila are known to be gregarious in the wild (Lazenby et al. 2007), we hypothesize that aggression occurred in our captive colony due to a lack of space or structural complexity that contributed to stress, culminating in aggression in susceptible females because the male cannot be evaded after mating (Noirot et al. 1975; Weber and Olsson 2008). Notably, there was no clear evidence that aggression was less likely to occur when the male was larger than the female, as has been observed in laboratory rats (*Rattus norvegicus*; Flannelly and Flannelly 1985). Given that injuries from aggressive interactions may reduce survival in the wild (and, therefore, reproduction), in addition to the potential link between stress and negative reproductive outcomes, such as decreased fertility (Xi et al. 2022) and maternal cannibalism (Poley 1974), a focus for future research should be to minimize sources of stress that may contribute to aggression during pregnancy. For a reintroduction design, this should translate to the provision of appropriate space and structural complexity in any areas where pookila will be held together, in addition to trials of various holding periods (Table 4). From a previous pookila reintroduction trial (Smith et al. 2022), we know that injuries and (suspected) pregnancies will occur in groups of eight adult pookila (of equal sex ratio) held together in an area of ~8.5 m^2^ for 10–15 nights (Smith K.J., ANU, Canberra, Australian Capital Territory, personal observation, November 2019).

### Facilitate free mate choice at release.

Heavier males were associated with pairing success and female-biased litters (which may be desirable for population establishment in the wild). The potential for the male to influence litter sex ratio is an emerging field of research, which we do not speculate on here (Douhard and Geffroy 2021). However, large body mass may have increased the attractiveness of the male, suggesting a role for female mate choice in reproductive output (Martin-Wintle et al. 2019). Free mate choice is easily facilitated in a reintroduction design with multianimal groups held together or released at the same time (Table 4). It is important that both large and small males are released together with females to avoid exerting artificial selection (Parrott et al. 2006). Further research on the phenotypes of pookila (including behaviors) and their relationship to reproductive success (and survival) in the wild is needed.

### Provide reproductive cues.

The presence of pups in the room was not associated with pairing success, but did increase the likelihood of aggression during pairing. This observation is consistent with evidence from other rodents that pup-related cues (e.g., smells) stimulate maternal aggression (Ferreira and Hansen 1986; Elwood et al. 1990). Given the link between aggression and pairing success, this suggests that pup-related cues (including sounds) might also promote breeding, but that in the ANU colony, the ultimate outcome of a pairing may be mediated by the negative effects of stress related to aggression. This hypothesis was supported by the observation that litter size is expected to be larger when pups are present in the breeding room (e.g., potentially promoting ovulation of more eggs or retention of more embryos by the female). We recommend experimenting with the provision of bedding from boxes containing breeding pairs, pregnant females, or pups, at release (Table 4).

Since the entire ANU colony was housed in wire-topped boxes in the same room, there was no opportunity to test for the potential influence of an olfactory, visual, or auditory signal-mediated ‘Bruce Effect’—a phenomenon first identified in laboratory mice where females terminate pregnancies when exposed to a new male (Bruce 1960; Zipple et al. 2021). However, the increased likelihood of pairing success when there are more pairs held together simultaneously in the same room accords with postulations that the presence of other females (Bruce 1960) or reproductive pheromones (Lambert et al. 2016) might keep animals in breeding mode. This hypothesis agrees with the peak in annual pairing success rate that occurred in 2019 (Fig. 5) before the colony size (and capacity for a large number of simultaneous pairings) was dramatically reduced due to COVID-19 lockdown restrictions. This suggests that there may be some benefit to the reproductive output of a group of pookila if a subset of the females is mated just prior to release (Table 4). The influence of pheromones on the reproductive output of Australian native rodents should be targeted for further research.

### Breed early and release effective pairs.

There was evidence to suggest that females first paired at a younger age would be less likely to be aggressive, without having any influence on the likelihood of pairing success. As such, we recommend that a familiarity with breeding be fostered in female pookila from an early age, prior to release (Table 4). In support of this approach, there is increasing evidence from a broad range of taxa (including rodents) that reproductive output is negatively impacted following extended periods without opportunity to breed; this consequence has been dubbed ‘use it or lose it’ (Penfold et al. 2014; Chung et al. 2018). However, we acknowledge that there may be practical limitations to this approach within a captive breeding program, and therefore also recommend trialing the release of recently weaned animals (i.e., before they are mature enough to be paired in captivity).

Increasing male age was associated with a lower probability that aggression signs would be observed during pairing, and was not negatively related to pairing success. We hypothesize that these relationships reflect an advantage of males having pairing experience (which itself was removed from the model due to correlation with male age). Therefore, where possible, we also recommend fostering a familiarity with pairing in males before their release (Table 4). While no trends regarding parity were evident for females, the greatest likelihood of producing pups may be achieved by pairing (or releasing together) a male and female that have previously been successful together (Table 4). Conversely, an unsuccessful pairing indicates that a pair may be incompatible. However, if subsequently re-paired and successful, a previously unsuccessful pairing was more likely to produce a male-biased litter than a new pairing. This may be related to the aging of the female, since older females are also expected to produce male-biased litters. As might be expected, older females were also more likely to suffer breeding complications and produce smaller litters, which highlights the importance of not limiting a release to older individuals (Table 4).

### Trial release of individuals with health conditions.

Our results indicated that females with health conditions were unlikely to reproduce. This suggests that any negative effects of seizures, freeze behavior, or metabolic conditions may extend to include impacts on the fertility of a female. A similar effect for males with health conditions was not evident, but the sample size for this variable was very small. However, there was some evidence for an absence of aggression in pairings where the male was found to be suffering a health condition, which suggests that they may be disinterested in mating or unattractive to the female (reinforcing the aforementioned hypotheses regarding female mate choice).

Further research is required to determine whether the health conditions we observed are hereditary, as has been recorded for seizures in some laboratory mice (Pesapane and Good 2009). Given that it is uncertain whether these health conditions would negatively impact fitness in the wild (e.g., individuals may never encounter a situation where a seizure is triggered), we suggest practitioners trial the release of individuals with a health condition (Table 4). If seizures have a negative impact on fitness, we anticipate that phenotype could be removed from a wild population by natural selection. By not immediately disqualifying individuals with a health condition, we could avoid inadvertent selection against the freeze behavior (i.e., a potentially beneficial antipredator response that may be confused with true seizures; Table 3), or other potentially beneficial genotypes and phenotypes held by these individuals.

### Generation number, diet, and inbreeding.

Increasing female generation number in the ANU colony was associated with pairing success until at least generation 4. This mirrors the increasing pairing success observed each year, peaking in 2019 when the number of simultaneous pairings and pups present in the same room was high, and the generation-4-females were less than 1 year old. A separate pookila breeding program (in the state of Victoria) observed the opposite effect—first and second generation females were more likely to have large litters, and had more litters in total (per female) than females from later generations (Lock 2004). This decrease in reproductive success with increasing generations in captivity has been recorded for other species, such as the Tasmanian devil (*Sarcophilus harrisii*; Farquharson et al. 2017). Given the collinearity between female generation number (mirroring time since colony opening) and diet (requiring the removal of the latter from multiple analyses), it is possible that any negative trends over the generations in our pookila colony were masked by the changes we made to the husbandry protocol over time, if those changes were, in fact, beneficial.

Based on experiments where laboratory mice that were provided high-fat diets tended to breed less readily, had significantly higher pup mortality, maternal cannibalism, or reduced male fertility, than mice on standard chow diets (Alexenko et al. 2007; Ghanayem et al. 2010; Bellisario et al. 2015), we believe the change we made to a low-fat diet in August 2017 could have benefited the reproductive output of pookila in the ANU colony (i.e., potentially contributing to a generational effect). More research is required to understand the potential influence of diet (and other husbandry factors) on reproductive output for the pookila. Although the potential negative effects of a high-fat diet may not be the same in the wild (Bomford 1987), as a precaution, we suggest that high-fat supplementary foods not be provided for long periods postrelease (Table 4).

If not a reflection of improved husbandry protocol, the generational effect may suggest that the pookila have adapted to captivity over time, potentially making them maladapted to the wild environment they are destined to return to (Frankham 2008). There was evidence to suggest a change in litter sex ratio between wild- and captive-born females. Aggression during pairing was less likely to be observed when the male was captive- rather than wild-born. Additionally, the lower overall pairing success observed following the COVID-19 lockdown bottleneck event suggests that inbreeding depression may have been affecting the reproductive output of the colony (though we also hypothesize that the reduced capacity for a large number of simultaneous pairings had a negative impact on pairing success rate, see above).

The lack of evidence for an effect of kinship or inbreeding coefficient might have been expected given that there were many zero-values for these variables (owing to our assumption that founders were unrelated and not inbred, our subsequently nonrandom pairing protocol, and the relatively shallow pedigree where most pairings occurred between individuals from generation 5 or less). Nonetheless, we included these variables to demonstrate the range of predictor categories that may be included in analyses of captive breeding data. We chose not to simulate genetic diversity in this case so that our recommendations for reintroduction design would be consistent with the pairing protocol used in the ANU colony. Conventional pedigrees are commonly used and still have value in the conservation genomics era (Rudnick and Lacy 2008; Galla et al. 2022). However, we acknowledge that the relatedness coefficients that could be calculated using our pedigree-derived estimates for kinship and inbreeding are likely to be underestimates of (albeit correlated with) that which could be obtained with genome-wide single nucleotide polymorphisms (Galla et al. 2020) and/or incorporating other data, such as point of collection and birth year (Hogg et al. 2019). Studies on the genetics of wild and captive pookila are required to aid the conservation of this species.

### Global applicability.

Making decisions for how to proceed with a reintroduction program can be challenging. Our analysis of the ANU pookila colony has demonstrated how the wealth of information in well-kept captive breeding records (typically used to improve breeding efficiency and maintain or improve the quality of individuals) can also be applied to decision-making during the design of a reintroduction, aided by the Translocation Tactics Classification System (Batson et al. 2015). An adaptive management approach is key; the recommendations we have made (Table 4) should be treated as hypotheses to be evaluated and refined through trials and experiments as part of the long-term ‘pathway to the wild’ for the pookila (Evans et al. 2022). It will also be important to balance the sustainability of remnant populations with the collection of founders that may be necessary to support optimal reproductive output in both the captive and reintroduced populations. The direct integration of knowledge derived from captive breeding with decision-making for reintroductions, as described here, will help address the uncertainties around the postrelease survival and reproduction of a species, which are common in reintroduction practice. Given that decades of research may be required to redress decline of a species (Santymire and Graves 2020), this process stands to benefit the conservation of both understudied and well-known species around the world.

## Supplementary Material

gyad056_suppl_Supplementary_Data_SD1

gyad056_suppl_Supplementary_Data_SD2

## Supplementary Data

**Supplementary Data SD1.**—Model selection tables (subsetted to models with ∆AICc ≤ 6) generated with the ‘dredge’ function in package ‘MuMIn’ (Bartoń 2022) from the models of pairing success, aggression, litter size, sex ratio, and breeding complications for the pookila (*Pseudomys novaehollandiae*) colony at the Australian National University.

**Supplementary Data SD2.**—Conditional averaged summaries from model averaging across all models with ∆AICc ≤ 6 for predictors of pookila (*Pseudomys novaehollandiae*) pairing success, aggression, litter size, sex ratio, and breeding complications at the Australian National University. The strength of evidence for an effect is given by ‘*P*,’ where ‘.’ indicates ‘weak evidence,’ ‘*’ indicates “moderate evidence,’ ‘**’ indicates ‘strong evidence,’ and ‘***’ indicates ‘very strong evidence’ (Muff et al. 2022).
